# *Glaesserella parasuis* Infection Modulates the Transcriptome of Porcine Peritoneal Mesothelial Primary Cells: Implications for Understanding Peritoneal Invasion Mechanisms

**DOI:** 10.3390/biology15070565

**Published:** 2026-04-01

**Authors:** Pu Guo, Jialong Fan, Yangfan Dong, Jiacheng Zhang, Qirong Lu, Chun Ye, Shulin Fu, Zhongyuan Wu, Yu Liu, Yinsheng Qiu

**Affiliations:** 1 Hubei Key Laboratory of Animal Nutrition and Feed Science, School of Animal Science and Nutritional Engineering, Wuhan Polytechnic University, Wuhan 430023, China; guopu@whpu.edu.cn (P.G.); 15393339613@163.com (J.F.); 13297953032@163.com (Y.D.); z139578@126.com (J.Z.); qirongluvet@whpu.edu.cn (Q.L.); yechun@whpu.edu.cn (C.Y.); shulinfu@whpu.edu.cn (S.F.); zhongywu@whpu.edu.cn (Z.W.); 2 Wuhan Engineering and Technology Research Center of Animal Disease-Resistant Nutrition, School of Animal Science and Nutritional Engineering, Wuhan Polytechnic University, Wuhan 430023, China

**Keywords:** porcine peritoneal mesothelial primary cells, *Glaesserella parasuis*, peritonitis, inflammation, transcriptome

## Abstract

Polyserositis is an important clinical feature of *Glaesserella parasuis* (*G. parasuis*, GPS) infection in pigs, typically presenting as peritonitis, pleuritis, pericarditis, meningitis, and arthritis, resulting in heavy economic losses in the swine industry. However, the current research on the pathogenesis of infectious peritonitis, particularly that caused by GPS, remains limited, and this condition has long been poorly reported in both clinical practice and research. In this study, we investigated the overall changes in gene expression in porcine peritoneal mesothelial primary cells (PPMCs) following a GPS infection using transcriptomics analysis. A total of 779 differentially expressed genes (DEGs) were identified after 12 h of infecting the PPMCs with GPS, resulting in 253 and 526 genes being upregulated and downregulated, respectively. Additionally, 220 DEGs, mainly involved in the NOD-like receptor signaling pathway, the TNF signaling pathway, and the metabolic pathway, were enriched in the KEGG analysis. These pathways were associated with the main DEGs (IL-1β, IL6, CCL5, CCL2 and NFKBIA), and their gene expression levels were verified through quantitative real-time fluorescence PCR (qRT-PCR). Moreover, oxidative phosphorylation, Salmonella infection, rheumatoid arthritis, and other regulating pathways were clustered together. Our results provide insights into the molecular mechanism underlying GPS-induced peritonitis in swine, identify novel therapeutic targets, and provide research direction for the control and prevention of GPS infections. These insights provide a foundational basis for advancing intervention and prevention approaches for this overlooked yet clinically significant manifestation of polyserositis.

## Simple Summary

*Glaesserella parasuis* causes serious infections and peritonitis in pigs, leading to significant economic losses, yet its disease-causing mechanisms are not fully understood. To investigate host cell responses to this infection, we analyzed the changes in gene activity in pig abdominal cells after infection. The results showed widespread changes in cellular gene activity, with many related to inflammation and energy metabolism pathways. These findings improve our understanding of the molecular processes through which this bacterium causes disease and provide potential targets and directions for developing new strategies to control and prevent these infections.

## 1. Introduction

The Gram-negative bacterium *Glaesserella parasuis* (*G. parasuis*, GPS, formerly *Haemophilus parasuis*, *H. parasuis*) is a member of the family *Pasteurellaceae* and the etiologic agent of Glässer’s disease in swine, with the typical characteristics of polyserositis, meningitis and arthritis, resulting in heavy economic losses in the swine industry [1,2,3]. GPS has been classified into at least 15 serovars to date, while approximately 20% of field isolates remain non-typeable even by conventional serological methods such as the agar diffusion test [2,4]. However, with the development of biological technology, an increasing number of virulence-related genes of GPS have been identified. Despite this progress, the virulence mechanism of infection remains unclear [5,6]. Thus, it is important to study the host inflammatory response induced by GPS, as it is a major bacterial respiratory pathogen in swine.

A recent study has shown that GPS frequently colonize arthrosis crevicular fluid in 20-year-old humans and can trigger a pro-inflammatory response, resulting in an enhanced production of IL-17 [7]. This indicates that GPS causes arthritis not only in swine but also in humans. Another study shows that in vivo changes in whole-genome DNA methylation patterns and gene expression profiles in porcine brains are an important molecular mechanism of GPS-induced meningitis [8]. While previous studies have elucidated certain mechanisms underlying GPS-induced arthritis and meningitis, the pathogenesis of polyserositis—particularly peritonitis—remains largely overlooked [9,10,11]. The peritoneum is a dynamic organ that orchestrates inflammatory responses, maintains fluid homeostasis, and prevents fibrosis. The dysregulation of these functions can lead to severe complications, including ascites, fibrous adhesions, and refractory peritonitis, posing significant therapeutic challenges [12,13]. Fibrinous peritonitis is a well-documented postmortem finding in GPS-infected pigs, with existing reports on polyserositis having primarily centered on clinical symptomatology. The main symptoms of peritonitis caused by GPS-infected pigs include the accumulation of moderate amounts of turbid and straw-colored serous effusion in the peritoneal cavity, which gradually progresses to fibrous exudate, ultimately forming an extensive fibrinosuppurative exudate that coats the serosal surfaces of the abdominal organs and forms focal adhesions [14,15]. However, the molecular mechanisms by which GPS elicits peritoneal inflammation remain to be systematically elucidated. Such investigations will not only advance our fundamental understanding of Glässer’s disease but also identify novel therapeutic targets and intervention strategies for this clinically significant manifestation.

Bacterial infections, including those caused by GPS, can lead to severe sepsis and polyserositis, often accompanied by secondary ascites. These conditions result in significant economic losses to the swine industry, with mortality rates reaching approximately 30% [16,17,18]. However, no systematic study concerning the mechanism of GPS-induced peritonitis has been published thus far. It is proposed that the specific signaling molecules and tissue-specific signaling factors from a given microorganism are involved in infections affecting the different tissues and organs [19,20]; however, the molecular mechanisms underlying GPS-induced peritonitis remain unclear. Therefore, an understanding of the differentially expressed genes (DEGs) that are involved in the peritonitis response may lead to a better understanding of the pathogenesis of GPS infection. Transcriptomic analysis has become a powerful tool for investigating host–pathogen interactions at the molecular level [21,22]. In this study, we employed RNA sequencing to profile the transcriptome of porcine peritoneal mesothelial primary cells (PPMCs) infected with the highly virulent *G. parasuis* SH016 strain (serovar 5) [10,23,24], with the goal of elucidating the host transcriptional response and the pathogenic mechanisms underlying GPS-induced peritonitis. These findings are expected to provide new targets and a direction for the control and prevention of GPS infection.

## 2. Materials and Methods

### 2.1. Bacterial Strain and Growth Conditions

The *G. parasuis* SH0165 strain, a highly virulent strain of serovar 5 isolated from a commercial pig with symptoms of fibrinous polyserositis, was used in our study [23]. This strain was cultured at 37 °C in tryptic soy broth (Difco, Lawrence, KS, USA), which was supplemented with nicotinamide adenine dinucleotide (Sigma, St. Louis, MO, USA) and fetal bovine serum (Gibco, Gaithersburg, MD, USA) [2].

### 2.2. Isolation and Culturing of Porcine Peritoneal Mesothelial Primary Cells

Three 30-day-old pigs were purchased from Wuhan COFCO Meat Product Co., Ltd. (Wuhan, China). The pigs were Duroc × Landrace × large white weaned piglets, weighing approximately 10 kg each, which all tested negative for *G. parasuis* antibodies (INGEZIM, Madrid, Spain). All the experimental animals were sacrificed at the end of the experiments. The study was approved by the Animal Care and Use Committee of Wuhan Polytechnic University, Wuhan, China (WPU202203001).

The process of separating the PPMCs is similar to that for porcine aortic vascular endothelial cells (PAVECs), as described in a previous article [24]. A piglet weighing approximately 10 kg was anesthetized by intramuscular injection of pentobarbital sodium behind the ear for 10–15 min. Following complete anesthesia, the piglet was euthanized by exsanguination. The abdominal area was disinfected with an alcohol swab, and a midline abdominal incision was made through the skin, muscle, and fat layers. The peritoneum was carefully exposed and gently removed after dissecting the surrounding adipose tissue. The collected peritoneal tissue was immediately placed in pre-chilled PBS (4 °C) and processed within 6 h. The removed peritoneal tissue was washed with PBS until no visible blood remained, digested with trypsin for 30 min, and neutralized with serum. After centrifugation at 2000 r/min for 15 min, the cell pellet was resuspended in DMEM/F-12 cell culture medium (20% serum) at 37 °C in a 5% CO_2_-humidified atmosphere for culture. The medium was replaced 12 h after seeding and then daily. The cells exhibited the characteristic cobblestone morphology that is expected under phase-contrast microscopy.

### 2.3. Transcriptome Design

Two treatment groups were established: a blank control group and a GPS model group, each with three biological replicates. The cells were initially seeded at a concentration of 10^6^ cells/mL. After 12 h of initial culture, the cells were counted, and the GPS model treatment group was exposed to a bacterial concentration of 10^8^ CFU/mL, corresponding to a multiplicity of infection (MOI) of 100, for 12 h. The blank control group received no bacterial treatment and was cultured under the same conditions. Following the 12 h infection period, the samples were collected for subsequent analysis.

### 2.4. Library Construction and Illumina Sequencing

The TRIzol reagent (ThermoFisher Scientific, New York, NY, USA) was used to extract the total RNA of the PPMCs, which was purified using the Dynabeads mRNA Purification Kit (ThermoFisher Scientific, New York, NY, USA). An Agilent Bioanalyzer 2100 (Agilent Technologies, Inc., Santa Clara, CA, USA) was used to assess the RIN number, which reflects the RNA integrity. For deep sequencing, only RNA with an RIN score of ≥7.0 and a 28S/18S ratio of ≥0.7 was selected.

The cDNA libraries were constructed with RNA isolated from the PPMCs using the TruSeq Stranded mRNA Library Preparation Kit (Illumina, San Diego, CA, USA).

### 2.5. Analysis of the Transcriptome Data

The transcriptome data were analyzed as described previously [10]. The RNA-seq data were processed following a standard pipeline. The raw sequencing reads were first assessed for quality using FastQC (version 0.11.5) [25]. The low-quality reads and adapter sequences were trimmed using Trimmomatic (version 0.36) to obtain clean reads. The cleaned reads were then aligned to the Sus scrofa reference genome (Sscrofa10.2, downloaded from Ensembl release 75) using Tophat2 (version 2.1.1) [26]. The gene expression levels were quantified as fragments per kilobase of transcript per million mapped reads (FPKM, Cufflinks v2.0.2) [27]. The differentially expressed genes (DEGs) were identified using DESeq version 1.18.0 [28], with thresholds of |log2FC| > 2 and q < 0.05 (FDR-adjusted *p*-value), indicating statistical significance.

A functional enrichment analysis was performed using DAVID version 6.7 [29] to identify significantly enriched Gene Ontology (GO) terms and KEGG pathways among the DEGs (q < 0.05). Additionally, the protein–protein interaction networks for the DEGs were analyzed using the STRING database [30].

### 2.6. Validation Using qRT-PCR

A total of 10 upregulated and 2 downregulated genes were chosen for the mRNA-level expression validation. The method for primer design was performed as previously reported (Table 1) [31], and GAPDH was used as the endogenous reference gene for normalization. The mRNA expression levels of 12 genes were determined by using SYBR Premix Ex Taq (TaKaRa, Dalian, China) in an ABI 7500 real-time PCR system (Applied Biosystems, Foster City, CA, USA). The thermal cycling conditions were as follows: denaturation at 95 °C for 15 s, annealing at 56 °C for 30 s, and extension at 72 °C for 30 s. For each treatment group, three biological replicates were included in each experiment, and the experiment was repeated three times. Their relative gene expression profiles were determined using the threshold cycle method, and their fold changes were calculated using the 2^−ΔΔCt^ formula [31].

### 2.7. Statistical Analysis

The *qRT-PCR* experimental data was expressed as the mean ± SD. The difference between the two groups was analyzed using the two-tailed Student’s *t*-test. Compared with the control group, * = *p* < 0.05 indicates a significant difference, while ** = *p* < 0.01 indicates a highly significant difference.

## 3. Results

### 3.1. Quality Analysis of Transcriptome Samples

**Figure 1:**
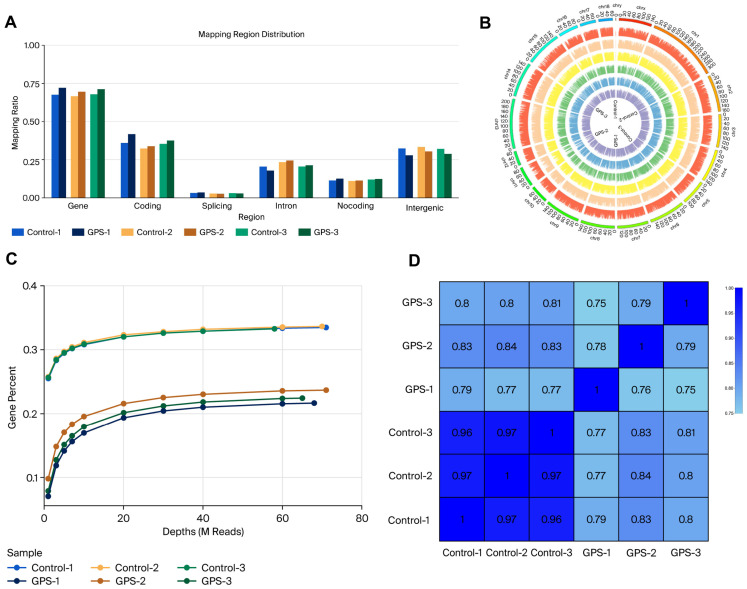
The quality analysis of transcriptome samples. (**A**) is the regional distribution of gene matching in the PPMCs; (**B**) is the genome coverage map of the PPMCs; (**C**) is the gene saturation analysis of the PPMCs; and (**D**) is the heatmap of the gene expression correlation in the PPMCs (Control-1, Control-2, and Control-3 are the control groups, and GPS-1, GPS-2, and GPS-3 are the PPMCs that are infected with GPS). Each cell represents the Pearson correlation coefficient based on the FPKM values, and the color scale indicates the correlation strength. The detailed sequencing quality scores and base distribution are shown in Figure S1.

RNA-seq was performed on six PPMC samples (three controls and three infected with *G. parasuis*) to profile the host transcriptomic response. The sequencing depth and alignment rates for each sample are summarized in Table S1. All the samples showed high mapping rates (>90%), indicating good data quality. In addition, all samples achieved high sequencing quality, with Q30 scores above 34.5, and a uniform base distribution (Figure S1). The analysis of the RNA-seq data quality showed that the percentages of reads mapping to intronic regions and intergenic regions were 72%, 30%, 5%, 25%, 12% and 26% for the six samples, respectively (Figure 1A). The genome coverage of the six samples was extremely high and almost complete (Figure 1B), and the gene detection reached saturation at approximately 60 million reads (Figure 1C). The sample correlation analysis revealed strong reproducibility between biological replicates, with the Pearson correlation coefficients exceeding 0.75 (Figure 1D). These results confirm that the sequencing data are of high quality and suitable for downstream differential expression analysis.

**Figure 2:**
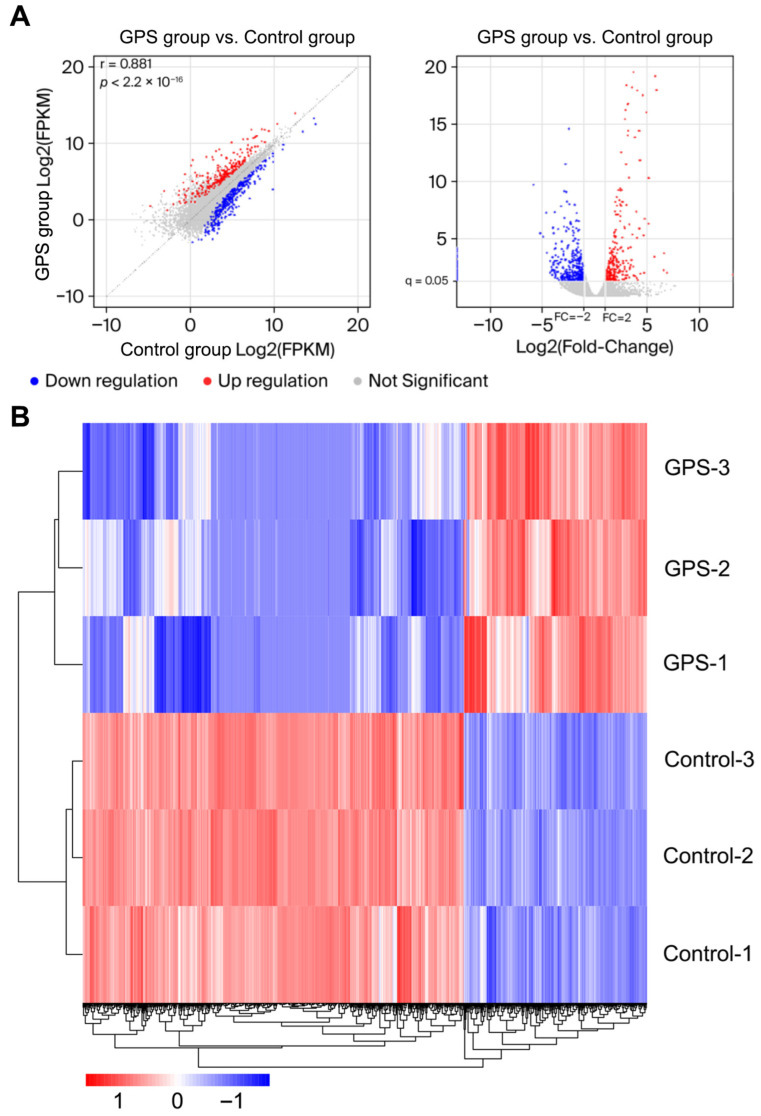
The analysis of the differentially expressed genes (DEGs). (**A**) The left panel of A shows a scatter plot showing the correlation between the control and infected samples. The right panel of A shows a volcano plot displaying the differentially expressed genes (blue indicates downregulated genes, gray represents non-differentially expressed genes, and red illustrates upregulated genes). The control group: Control-1, Control-2, and Control-3 represent the control groups. The GPS group: GPS-1, GPS-2, and GPS-3 represent the PPMCs infected with GPS. (**B**) The heatmap of differential expressions in the PPMCs (Control-1, Control-2, and Control-3 indicate the control groups, and GPS-1, GPS-2, and GPS-3 represent the PPMCs that are infected with GPS).

To assess the reproducibility and similarity between the samples, we analyzed the gene expression profiles of the PPMCs. As shown in the scatter plot (left part of Figure 2A), most points between the control group and the *G. parasuis*-infected group were concentrated near the diagonal, indicating a strong correlation and high similarity between the biological replicates.

### 3.2. Identification of DEGs in PPMCs

The PPMCs infected with *G. parasuis* showed a greater degree of differential expression. A total of 253 upregulated and 526 downregulated genes were significantly altered (≥2-fold change, q < 0.05), as shown in the volcano plot (right part of Figure 2A). Cluster software on R (version 4.0.5) was used to compare DEGs between the two groups and conduct cluster analysis. The upregulated, downregulated, and non-differentially expressed genes are shown in red, blue, and white, respectively (Figure 2B, Table S2), and show similar results to the gene scatter plot.

### 3.3. GO Enrichment and KEGG Enrichment Analysis

**Figure 3:**
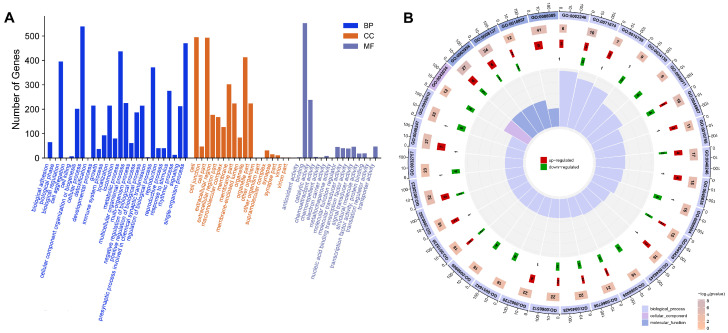
The functional analysis of the GO categories. (**A**) The x-axis displays the categories, and the y-axis displays the number of genes in the categories. The loop graph of the top 30 DEGs (**B**).

Bioinformatics tools: GO classification was performed to enrich and analyze the functional characteristics of the 779 DEGs identified after the host cell was infected with GPS (Figure 3A, Table S3). The metabolic process, signal transduction, biological regulation, cellular process, single-organism process, and bioprocess regulation were the most abundant categories in the biological process group, followed by the transport process and the immune system process. In terms of the cellular component group, the most abundant categories were cellular component and organelle. Moreover, antioxidant activity, transporter activity, binding, catalytic activity, and regulator function were enriched in the molecular process. The first 30 enriched genes are mainly involved in the following biological activities: chemokine activity, NADH dehydrogenase activity, lymphocyte chemotaxis, interleukin-2 production, and ventricular cardiac muscle tissue morphogenesis (Figure 3B).

Meanwhile, the GO enrichment items such as cell death, cardiovascular system development, circulatory system development, programmed cell necrosis, cell morphological and structural changes, cell apoptosis, MAPK cascade reaction, and protein phosphorylation signal transduction were also matched through a significant comparison. It was determined that these terms matched the key enrichment items of the cells that were mainly involved in cell death, apoptosis, MAPK cascade, protein phosphorylation, and other GO enrichment items, indicating that cell death and apoptosis may be associated with the phosphorylation activation of the MAPK cascade. This notion was directly supported by the specific expression patterns of the key regulatory genes during endothelial apoptosis, including the upregulation of pro-apoptotic factors such as ANGPTL4, TNFAIP3, and ICAM1, as well as MAPK4 itself, coupled with the downregulation of the protective gene GAS6.

**Figure 4:**
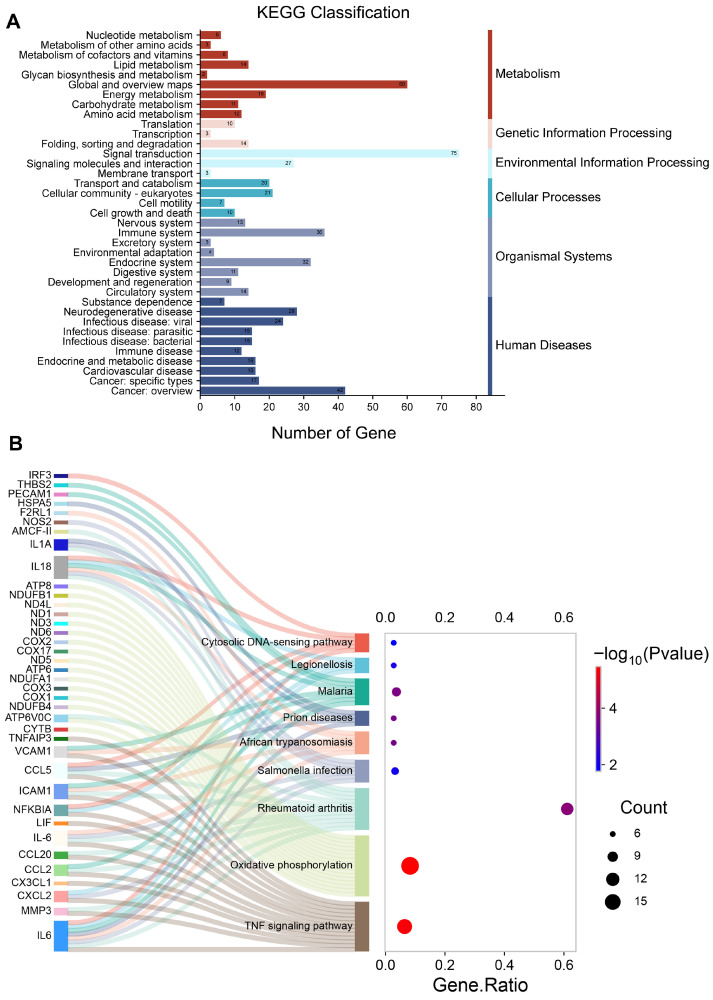
The KEGG pathway classification analysis (**A**). The DEGs involved in the main pathways (**B**).

Through the KEGG pathway enrichment analysis, we found that the DEGs were mainly involved in the immune system, amino acid metabolism, the endocrine system, signaling molecules and interaction, signal transduction, cell growth and death, and transport and catabolism, while other DEGs were associated with metabolism and cell components (Figure 4A, Table S4). Furthermore, the main metabolic and signaling pathways were the TNF signaling pathway, the NOD-like receptor signaling pathway, the oxidative phosphorylation pathway, the Salmonella infection pathway, the rheumatoid arthritis pathway, as well as other regulating pathways (Figure 4B).

### 3.4. STRING Analysis of the Association Among DEGs of the Main Pathways

**Figure 5:**
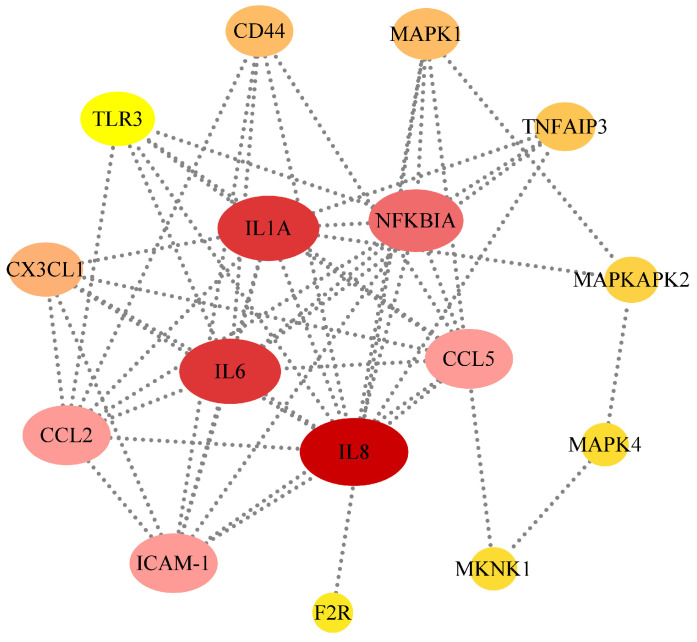
The associations between the proteins were determined by using STRING analysis.

To predict the interaction network, STRING analysis was performed to obtain the interaction network between the proteins encoded by the DEGs. In our study, the complex associations between the 16 DEGs that participated in the main pathways were analyzed according to the STRING v10 database. Most of the DEGs were closely related to each other, showing a coordinated network of interactions (Figure 5). Through the analysis of protein–protein network interactions, it was speculated that NFKBIA-mediated inflammatory injury was the main response to peritonitis caused by GPS.

### 3.5. Real-Time Polymerase Chain Reaction (PCR) Verification of DEGs

**Figure 6:**
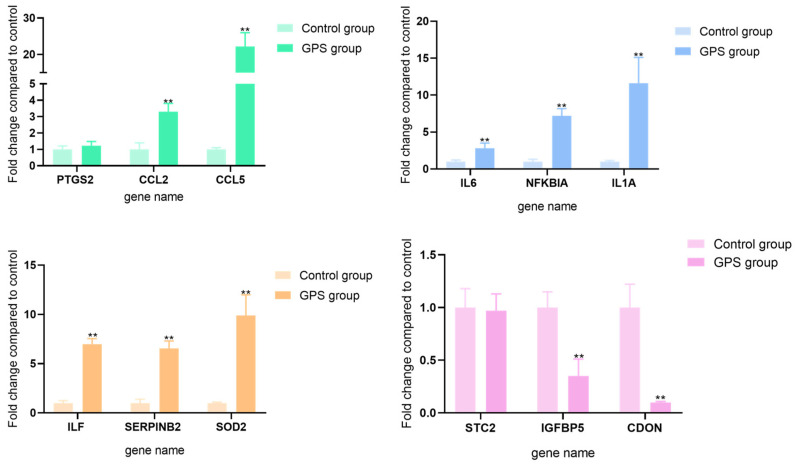
The relative quantification of the DEGs for verification. GPS: *G. parasuis*. ** = *p* < 0.01 indicates a highly significant difference.

A real-time polymerase chain reaction (RT-PCR) was performed to detect the expression profiles of 12 genes that were chosen for the verification of the DEG from the five functional categories. The 12 chosen genes displayed similar expression levels to the transcriptome data (Figure 6). Compared with the control group, the expression levels of CCL2, CCL5, IL6, NFKBIA, IL-1A, LIF, SERPINB2, and SOD2 were significantly increased, while the expression levels of IGFBP5 and CDON were significantly downregulated. Additionally, PTGS2 showed an upward (non-significant) trend while STC2 displayed a downward (non-significant) trend, according to qPCR.

## 4. Discussion

Although extensive research has focused on the pathogenesis of GPS, the molecular mechanisms underlying its induction of polyserositis remain largely unexplored. Peritonitis represents a consistently observed yet mechanistically overlooked component of Glässer’s disease, and the specific pathways by which GPS interacts with peritoneal cells to trigger inflammation and tissue injury have not been elucidated. To address this knowledge gap, we employed the PPMCs as an in vitro model, which closely recapitulates the cellular components of the serosal microenvironment. Following 12 h of exposure to GPS in vitro, demonstrating the responses of host cells to GPS, global transcriptional profiling was performed to characterize the early host response. This approach enables the identification of the key genes and pathways involved in peritoneal inflammation and provides a foundation for understanding the molecular basis of GPS-induced peritonitis. From a GO enrichment perspective, the results of the DEG analysis of the transcriptomic mRNA data showed that the main effects of GPS on the PPMCs involved an inflammatory response, an activation of the toll-like receptor signaling pathway, the regulation of protein kinase activity, chemotaxis of eosinophils, the apoptotic process, and interleukin production. Additionally, the analysis of the matched key enrichment entries reveals two main categories: one is the expression of inflammatory genes, such as those involved in toll-like receptor activation, eosinophil chemotaxis, and interleukin production, while the other is the emergence of apoptotic processes, such as the regulation of protein kinase activity and apoptotic processes. This suggests that, after a GPS infection in PPMCs, apoptosis occurs in addition to the inflammatory response; due to the regulation of protein kinase activity and the MAPK cascade response seen in the focus-matching entry, it can be speculated that the phosphorylation of MAPK signaling pathway-related proteins may have induced apoptosis. In addition, the KEGG enrichment analysis showed that the TNF signaling pathway, the NOD-like receptor pathway, and the metabolic pathway are the main signaling pathways, with the main DEGs (IL-1β, IL6, CCL5, CCL2 and NFKBIA) observed at the gene expression level.

During the pathological process of peritonitis, NOD-like receptors (NLRs) and the tumor necrosis factor (TNF) signaling pathway are the core mechanisms driving the inflammatory response. After NOD1/NOD2 recognize the bacterial peptidoglycan, they activate NF-κB, promoting the transcription of pro-inflammatory factor genes, including IL-1β/IL-18, which directly damage the intestinal mucosal barrier [32,33]. TNF-α expression increases in cases of peritonitis, which can also increase vascular permeability, inhibit the expression of tight junction proteins, and exacerbate the development of peritonitis [34]. These two pathways are closely interconnected: the NOD-like receptor pathway induces the production of TNF-α, which can enhance the NLRP3 expression, forming a positive feedback loop and jointly promoting neutrophil infiltration, tissue necrosis, and even sepsis progression. The interventions targeting these pathways are becoming potential therapeutic directions.

As members of the chemokine system, CCL2 and CCL5 could induce the migration and recruitment of endothelial cells, T cells, dendritic cells, eosinophils, vascular smooth muscle cells (VSMCs), and mast cells in vitro [35,36,37]. Consistent with these functions, elevated levels of CCL2 and CCL5 are widely observed in diverse inflammatory conditions, where they contribute to leukocyte infiltration and disease progression. In the present study, both the transcriptomic analysis and the gene expression verification results showed that CCL2 and CCL5 were highly expressed in the GPS-infected cells, indicating that GPS caused an inflammatory response in the PPMCs, which is a kind of response that is typically ignored when studying GPS infection. In addition to chemokines, pro-inflammatory cytokines play a key role in infectious and non-infectious inflammatory diseases. The typical pro-inflammatory cytokines, such as IL-1β, IL-6, TNF-α, IL-8, IL-12, IFN-γ, and IL-18, are mainly produced by activated macrophages and are involved in the upregulation of inflammatory responses [38]. These cytokines play a role in containing and resolving inflammatory foci by activating local and systemic inflammatory responses. IL-1 β and IL-6 are mainly released by monocytes, macrophages and non-immune cells in response to cell injury, infection, invasion and inflammation, and are more sensitized to bacterial infections [39]. As a member of the NF-κB inhibitory family, another inflammatory factor, NFKBIA, inhibits the activity of the dimeric nuclear NF-κB/REL complex and captures REL dimers in the cytoplasm by masking its nuclear localization signal [40]. An increased NFKBIA expression indicates the activation of a protective feedback mechanism in response to inflammation, which serves to regulate the NF-κB pathway activity [41]. Our results were similar to the description above and confirmed this mechanism.

Therefore, we speculate that CCL2 and CCL5 may play key roles as biomarkers, as they participate in the cellular damage process during a GPS infection. Thus, we aim to develop CCL2 and CCL5-based molecular targets to control GPS infection. Furthermore, in accordance with the anti-inflammatory effects on the NF-κB pathway, we suggest that NFKBIA activation may protect against the onset of vascular damage induced by GPS and may have a beneficial regulatory effect on environmental stress. However, the underlying mechanism of this effect needs to be further studied.

## 5. Conclusions

In conclusion, this transcriptomic analysis reveals substantial transcriptional reprogramming in the PPMCs infected with *G. parasuis* SH0165, with the differentially expressed genes significantly enriched in the pathways that were associated with inflammatory response, tight junction injury and cell survival. These findings provide a foundation for understanding the molecular mechanisms underlying GPS-induced peritoneal inflammation and injury. While our data offer new insights into the early host transcriptome response to GPS, the study’s limitations must be considered. The use of an in vitro system cannot fully replicate the complex peritoneal immune environment. Future studies incorporating time course analyses, additional strains, and functional validation will be essential to fully elucidate the complex host–pathogen interactions underlying Glässer’s disease and provide effective prevention and control strategies for swine.

## Figures and Tables

**Table 1:** The primers for the validation of transcriptome genes.

Genes	Primers (5′–3′)		Products (bp)
*β-actin*	Forward	TGCGGGACATCAAGGAGAAG	216
	Reverse	AGTTGAAGGTGGTCTCGTGG	
*IL-6*	Forward	GCAGACTGAGCCTTAGACATCC	110
	Reverse	CCCACCCTCCAACAAAGATTT	
*PTGS2*	Forward	CCCTTGAAGTGGGTAAGTATGT	122
	Reverse	TGGGTCTGGTTAACTTCTCTCT	
*SERPINB2*	Forward	GAGGACACGATGGCTGAAGATG	189
	Reverse	AGAGGCTTGATGGAACACTTGG	
*CCL2*	Forward	TCGATGCAGCGGCTGATGA	139
	Reverse	GGTGGCTTATGGAGTCCTGGA	
*LIF*	Forward	CCTCCACCATCACCACCTTGT	108
	Reverse	TGCCTCCAACTCCTGCTGTC	
*SOD2*	Forward	CGTGGAGGAGAAGTACCAGGAG	230
	Reverse	CGACGGATACAGCGGTCAACT	
*NFKBIA*	Forward	GGTGTCGCTCTTGTTGAAGTGT	106
	Reverse	CTGCTGTATCCGAGTGCTTGG	
*IL1A*	Forward	GCTCTCTTCCTGCTCACCAACT	296
	Reverse	TGCTGCCATCACCACACTGT	
*STC2*	Forward	GTGAACCTGCTGCTGACCTGTG	287
	Reverse	TGGGCGATTGGGTGGCTCTT	
*CDON*	Forward	GCTGACGGAGACTCTGTGACT	291
	Reverse	CGCAAGGACAACTTCTGACCAT	
*IGFBP5*	Forward	GCCGTGAAGAAGGACCGCAGAA	289
	Reverse	ACTTGTCCACGCACCAGCAGAT	
*CCL5*	Forward	TGGCAGCAGTCGTCTT	244
	Reverse	CTCCATCCTAGCTCAACTC	

## Data Availability

All the data are presented in the article.

## Supplementary Materials

The following supporting information can be downloaded at https://www.mdpi.com/article/10.3390/biology15070565/s1, Figure S1. Sequencing quality metrics. (A) Sequencing quality scores (Phred score) across read positions for control and *G. parasuis*-infected samples. (B) Base composition distribution. Table S1. Statistics of preprocessing results of PPMC data. Table S2. Full list of differentially expressed genes. Table S3. Full GO enrichment results. Table S4. Full KEGG enrichment results.

## Abbreviations

The following abbreviations are used in this manuscript:

CCL2/MCP-1	monocyte chemoattractant protein-1
CCL5	C-C motif chemokine 5
CDON	cell adhesion associated
DEGs	differentially expressed genes
IGFBP5	insulin-like growth factor-binding protein
IL1A	interleukin-1 alpha
IL-6	interleukin-6
LIF	leukemia inhibitory factor; SOD2
NFKBIA	nuclear factor-kappa-B inhibitor alpha
PAVECs	porcine aortic vascular colorectal cells
PPMCs	porcine peritoneal mesothelial primary cells
PTGS2	prostaglandin-endoperoxide synthase 2
qRT-PCR	quantitative real-time fluorescence PCR
SOD2	superoxide dismutase 2
STC2	recombinant stanniocalcin 2
