# Supplementary material for: Glaesserella parasuis Infection Modulates the Transcriptome of Porcine Peritoneal Mesothelial Primary Cells: Implications for Understanding Peritoneal Invasion Mechanisms
Source: Biology (Basel). 2026 Apr 1;15(7):565. doi: 10.3390/biology15070565 (PMC13072060; doi:10.3390/biology15070565)
Supplement: Supplementary file 1 [file biology-15-00565-s001.zip › Figure S1 .pdf]

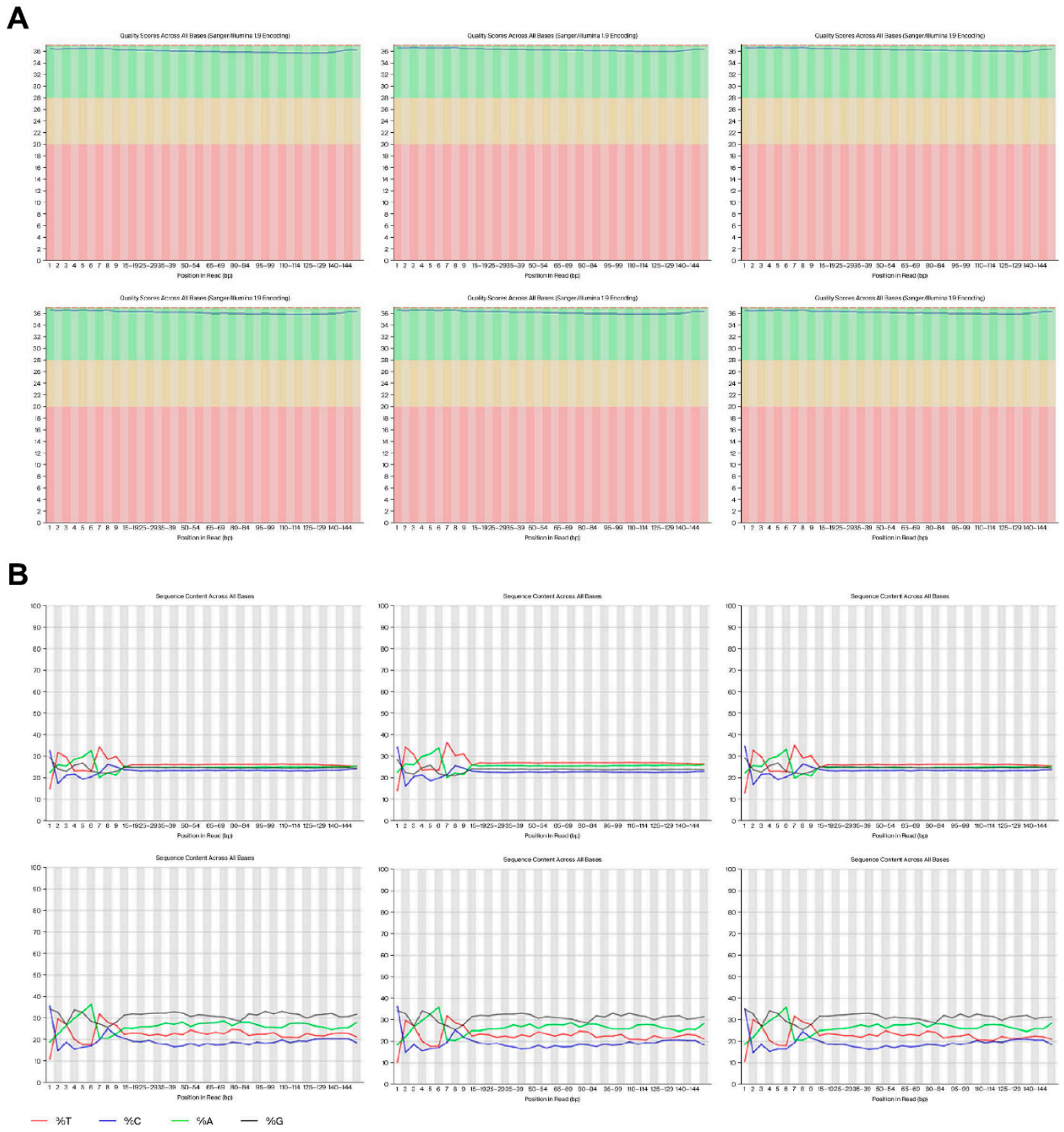


Figure S1. Sequencing quality metrics.

(A) Sequencing quality scores (Phred score) across read positions for control and G. parasuis-infected samples. (B) Base composition distribution.
