# Supplementary material for: Glaesserella parasuis Infection Modulates the Transcriptome of Porcine Peritoneal Mesothelial Primary Cells: Implications for Understanding Peritoneal Invasion Mechanisms
Source: Biology (Basel). 2026 Apr 1;15(7):565. doi: 10.3390/biology15070565 (PMC13072060; doi:10.3390/biology15070565)
Supplement: Supplementary file 1 [file biology-15-00565-s001.zip › Table S1 Statistics of preprocessing results of PPMC data.pdf]

Table S1 Statistics of preprocessing results of PPMC data

Sample ID	Raw reads	Clean reads	Clean ratio
Control	78194478	74034672	94.68%
	75116804	72254033	96.19%
	63853928	60692158	95.05%
GPS	72936100	71064674	97.43%
	76872440	74443960	96.84%
	71694482	68757075	95.90%

Note: Clean ratio=(Clean reads/Raw reads)%
